# Krüppel-like factor 7 deficiency causes autistic-like behavior in mice via regulating Clock gene

**DOI:** 10.1186/s13578-022-00903-6

**Published:** 2022-10-07

**Authors:** Hui Tian, Yanwen Jiao, Mingyue Guo, Yilin Wang, Ruiqi Wang, Cao Wang, Xiongbiao Chen, Weiming Tian

**Affiliations:** 1 School of Life Science and Technology, Harbin Institute of Technology, Harbin, 150080 China; 2 Department of Mechanical Engineering, University of Saskatchewan, Saskatoon, SK S7N 5A9 Canada

**Keywords:** ASD, klf7, Clock gene, Circadian rhythm, Melatonin

## Abstract

**Background:**

Krüppel-like factor 7 (klf7), a transcription factor in the nervous system to regulate cell proliferation and differentiation, has been recently identified as a causal gene for autism spectrum disorder (ASD), but the mechanism behind remains unknown.

**Result:**

To uncover this mechanism, in this study we characterized the involvement of klf7 in circadian rhythm by knocking down klf7 in N2A cells and examining the rhythmic expression of circadian genes, especially Clock gene. We constructed klf7^−/−^ mice and then investigated into klf7 regulation on the expression of rhythm genes in vivo as well as the use of melatonin to rescue the autism behavior. Our results illustrated that circadian rhythm was disrupted in klf7 knockdown cells and that klf7^−/−^ mice showed autism-like behavior. Also, we found that Clock gene was downregulated in the brain of these klf7^−/−^ mice and that the downstream rhythm genes of Clock were disturbed. Melatonin, as a circadian regulation drug, could regulate the expression level and amplitude of rhythm genes in klf7 knockout cells and further rescue the autistic behavior of klf7^−/−^ mice.

**Conclusion:**

Klf7 deficiency causes ASD by disrupting circadian rhythm related genes to trigger rhythm oscillations. To treat ASD, maintaining circadian homeostasis is promising with the use of melatonin.

## Introduction

Krüppel-like factor 7 (klf7) is a transcription factor in the nervous system to regulates cell proliferation and differentiation [1]. Klf7 has been proposed as a candidate gene for autism spectrum disorder (ASD) with the results that patients with deletion in 2q33.3q34, where klf7 is located, show autistic traits [2–6]. More recently, Powis et al., reported 4 unrelated individuals with de novo mutations in klf7 shared similar clinical traits, including developmental delay/intellectual disability, hypotonia, feeding/swallowing issues, psychiatric features, and neuromuscular symptoms [7]. In addition, rare de novo non-coding variants in this gene have been observed in ASD probands [8, 9]. These studies suggest a possible link between klf7 mutation and ASD. In our recent study, klf7 was confirmed to be an autism-related gene by constructing Nestin-Cre conditional knockout mouse model, though the mechanism behind remains unknown [10].

Klf7 was identified as a regulator related to suprachiasmatic nucleus function and could enrich rhythm genes in mouse suprachiasmatic nucleus [11, 12]. In this regard, we also discovered that target genes of both human klf7 and mouse klf7 were significantly enriched in circadian rhythms [10], indicating that klf7 is involved in circadian rhythm. More evidences have been recently collected to support the strong link between dysregulated circadian rhythm and ASD. Researches in cognitive and developmental psychology have discovered the important role of rhythm in the early development of social interaction [13] and impaired circadian clock network causes susceptibility to ASD [14, 15]. Circadian rhythm is regulated by clock-controlled genes (CCGs), variations in CCGs were considered to affect physiological functions, which may lead to disease susceptibility [16]. Investigations of circadian rhythm related genes showed that Clock, Per1, Per2, Per3, Npas2, Bmal1, Cry1, Cry2 and Dbp mutated frequently in ASD individuals [17, 18]. Also, researches suggested that abnormal CCGs had a significant impact on the development of ASD, especially for sleep–wake rhythm and communication disorders [19–23]; and that circadian clock genes played a significant role in ASD [24], suggesting the link between circadian rhythm and ASD. Based on the above discussion, in this study we hypothesized that klf7 could contribute to ASD via disrupting circadian rhythm, and tested by investigating into the klf7 regulation on the expression of rhythm genes in vivo. Furthermore, we demonstrated that the use of melatonin could rescue the autism behavior.

## Results

### Klf7 is involved in regulating the circadian rhythm-related genes

**Fig. 1:**
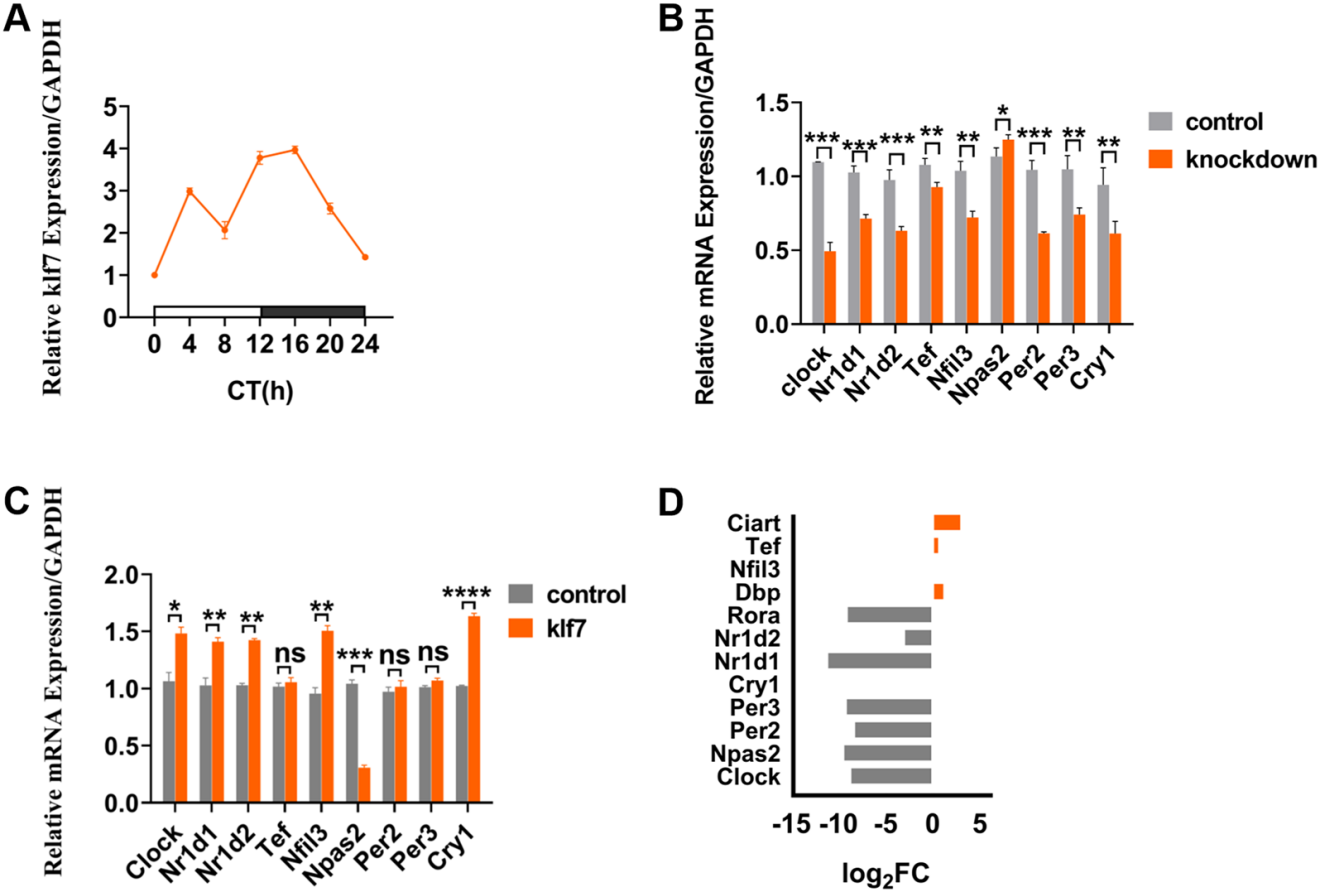
Regulation of circadian rhythm genes by klf7. **A** qPCR analysis of diurnal rhythm expression of klf7 in N2A cells. Peak presents at CT16 and nadir presents at CT0 (n = 4 independent experiments). **B** qPCR analysis of the expression of circadian rhythm genes induced by klf7 knockdown in N2A cells. Shown is fold change induced by klf7 knockdown (n = 4 independent experiments). **C** qPCR analysis of the expression of circadian rhythm genes induced by klf7 overexpression in N2A cells. Shown is fold change induced by klf7 (n = 4 independent experiments). **D** RNA-seq analysis of the expression of circadian rhythm genes in klf7 knockdown human brain organoid model compared with control group. Shown is log2(fold change) induced by klf7 knockdown (n = 2 independent experiments). The data are presented as the mean ± SEM. Statistical analysis (P^*^ < 0.05, P^**^ < 0.01 and P^***^ < 0.001) between control group and klf7 knockdown group in (**B**) were performed by unpaired t test. Statistical analysis (P^*^ < 0.05, P^**^ < 0.01, P^***^ < 0.001, P^****^ < 0.0001 and ns: no significance) between control group and klf7 group in (**C**) were performed by unpaired t test

We analyzed the expression of klf7 in N2A cells and obtain the diurnal rhythm (Fig. 1A), which peaked at CT16 and nadired at CT0 (CT0 is the onset at the lighting period). In our previous study [10], we found that the target genes of klf7 were significantly enriched in circadian rhythms. In order to confirm the effects of klf7 on circadian rhythm genes, we knocked down klf7 level in N2A cells with a lentiviral short hairpin RNA (shRNA). Meanwhile, we also transiently transfected klf7 gene into N2A cells. qRT-PCR results confirmed that klf7 could regulate the expression of some circadian rhythm genes (including Clock, Nr1d1, Nr1d2, Nfil3 and Cry1) (Fig. 1B, C). Organoids can simulate early brain development in vitro, which is necessary for the investigation of neurodevelopmental disorders at many developmental stages [25–27]. We knocked down klf7 with shklf7 in human brain organoid model, which was derived from hiPSCs (cell name: DYR0100, serial no.: SCSP-1301, National Collection of Authenticated Cell Cultures), and found that the expression level of rhythm genes was also disrupted, further supporting that klf7 was involved in the regulation of rhythm genes (Fig. 1D). These data suggest that klf7 may play an important role in the regulation of circadian rhythm genes.

### Klf7 binds to Clock gene and forms a feedback loop with Clock gene

**Fig. 2:**
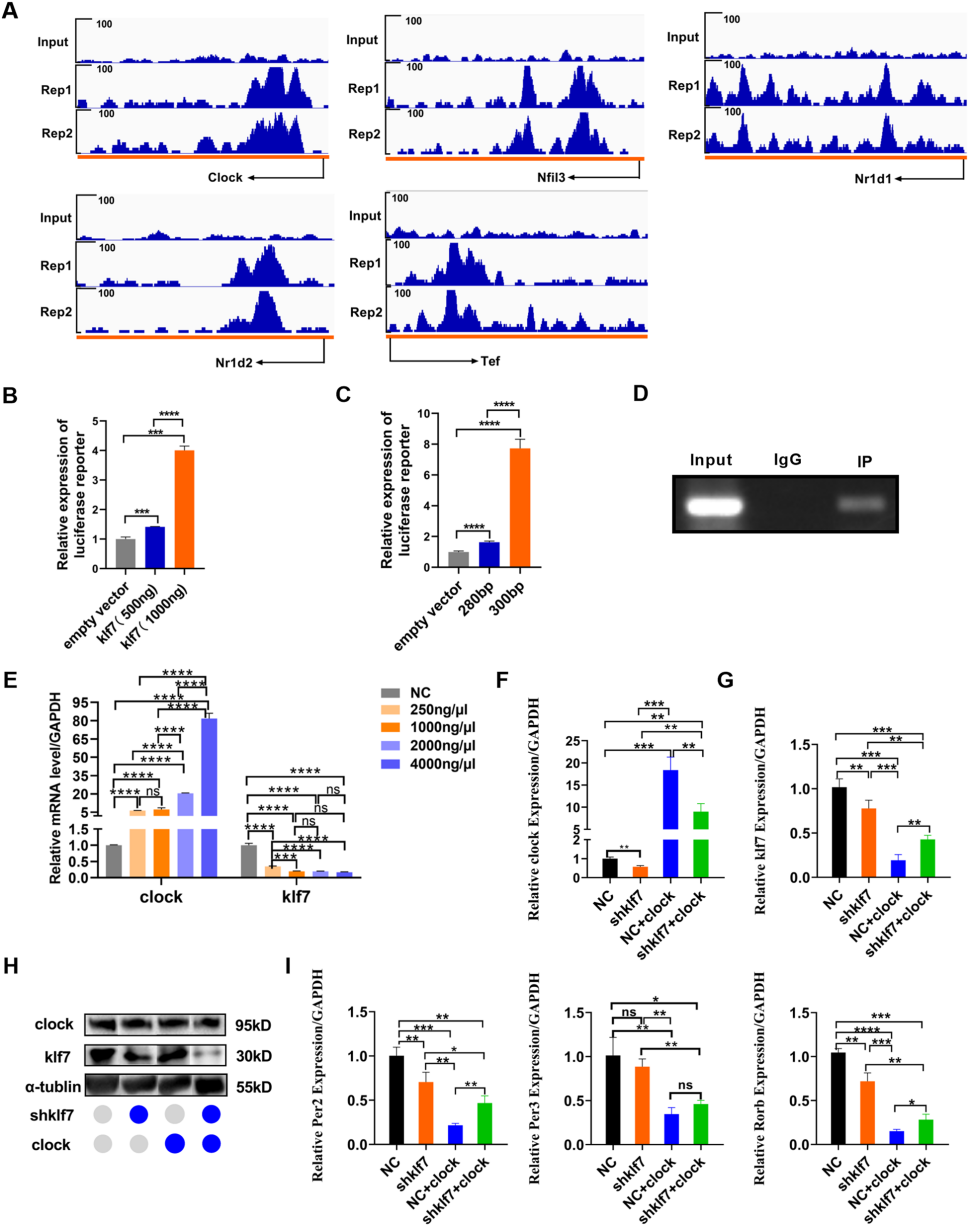
Klf7 binds to Clock gene and forms a feedback loop with Clock gene. **A** Binding peaks for circadian rhythm genes, which were identified in two biological replicates by Chip-seq. **B** Luciferase reporter assay of Clock promoter in the presence of klf7. Klf7 positively regulates Clock gene transcription in a concentration dependent manner (n = 4 independent experiments). **C** Transcriptional analysis of Clock truncated promoter quantified by luciferase reporter assay. Specific 300 bp sequence of Clock promoter can be positively targeted by klf7 (n = 4 independent experiments). **D** PCR primers that can amplify the specific 300 bp sequence of Clock gene promoter in IP group (n = 4 independent experiments). **E** Clock gene inhibits klf7 expression in turn (n = 4). **F** Clock mRNA levels measured by qPCR in NC group, shklf7 group, NC and Clock group, shklf7 and Clock group (n = 4). **G** klf7 mRNA levels measured by qPCR in NC group, shklf7 group, NC and Clock group, shklf7 and Clock group (n = 4). **H** Protein levels of klf7 measured by western blot. **I** mRNA levels of Per2, Per3 and Rorb measured by qPCR in NC group, shklf7 group, NC and Clock group, shklf7 and Clock group (n = 4). The data are presented as the mean ± SEM. Statistical analysis (P^***^ < 0.001 and P^****^ < 0.0001) among empty vector group, klf7 (500 ng) group and klf7 (1000 ng) group in (**B**) were performed by one-way ANOVA test. Statistical analysis (P^****^ < 0.0001) among empty vector group, 280 bp group and 300 bp group in (**C**) were performed by one-way ANOVA test. Statistical analysis (P^***^ < 0.001, P^****^ < 0.0001 and ns: no significance) among NC group, 250 ng/μL group, 1000 ng/μL group, 2000 ng/μL group, and 4000 ng/μL group in (**E**) were performed by one-way ANOVA test. Statistical analysis (P^*^ < 0.05, P^**^ < 0.01, P^***^ < 0.001 and ns: no significance) among NC group, shklf7 group, NC + clock group and shklf7 + clock group in (**F**, **G)** and (**I**) were performed by one-way ANOVA test

To verify the transcriptional regulation of klf7 on circadian rhythm genes, we performed ChIP-seq assays in N2A cells with the results showing that klf7 binded to Clock, Nr1d1, Nr1d2, Nfil3 and Tef (Fig. 2A). Clock gene is a core transcription factor in the circadian rhythm system, which forms a transcription-translation auto-regulatory feedback loop with Bmal1 and its target genes Per, Cry and Nr1d1. Target genes accumulate rhythmically and form a suppressor complex that interacts with Clock and Bmal1 to inhibit transcription [28]. Considering the significant influence of klf7 on Clock gene expression level and the core role of Clock in circadian system, we selected Clock gene to examine the relationship between circadian gene and ASD. The results from the Luciferase reporter gene assay confirmed that klf7 positively regulated the transcription of Clock gene in a dose dependent manner (Fig. 2B). The promoter of Clock gene was truncated into a 300 bp sequence and a 280 bp sequence, with the results demonstrating that specific 300 bp sequence could be positively bound by klf7 (Fig. 2C). PCR primers could amplify the 300 bp sequence of Clock gene promoter in IP group (Fig. 2D). These results suggest that klf7 can regulate Clock gene transcription.

The circadian rhythm system consists of transcriptional activators and transcriptional repressors, which can be assembled into feedback loops [29]. To determine whether there is such a transcriptional feedback loop between klf7 and Clock gene, we transiently transfected Clock gene in N2A cells by a dose dependent manner and found that Clock could inhibit klf7 expression, forming a negative regulatory loop (Fig. 2E). In order to verify the effect of increased Clock level on downstream rhythm genes, we selected 250 ng Clock plasmid for transient transfection of normal cells and klf7 knockout cells; qPCR was used to examine the expression efficiency after 48 h. Our results showed that high expression of Clock gene was observed in both normal cells and klf7 knockout cells (Fig. 2F). However, the expression level of klf7 was significantly decreased in both normal cells and klf7 knocked out cells after clock overexpression (Fig. 2G), and the changes in protein level were consistent with mRNA result (Fig. 2H). Also, we found that the levels of Per2, Per3 and Rorb were significantly downregulated in klf7 knockout cells. Increasing Clock level did not improve these genes expression levels, but caused them significantly decreased (Fig. 2I). We speculated that this was caused by Clock inhabiting klf7 expression, being associated with the regulation of klf7 on rhythm genes.

### Klf7 knockdown cells demonstrate dysregulated circadian phenotypes

**Fig. 3:**
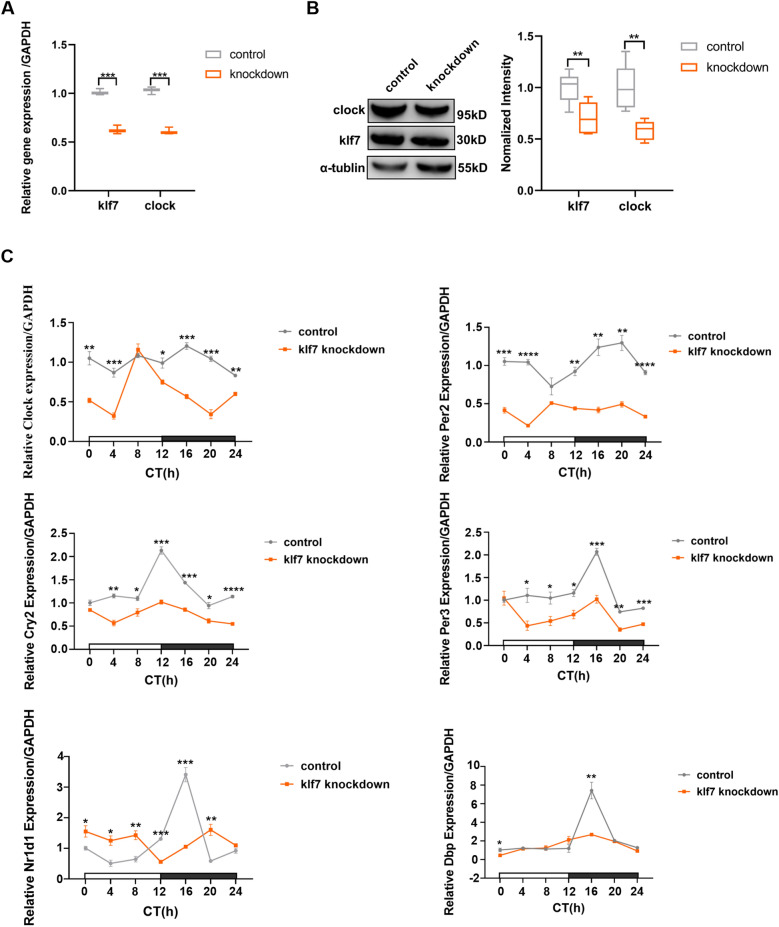
Klf7 influences rhythmic expression of core clock genes. **A** Klf7 and Clock mRNA levels measured by qPCR in klf7 knockdown cells. The Clock mRNA level was decreased significantly compared with control group (n = 4). **B** Clock protein levels measured by western blot in klf7 knockdown cells. Clock protein levels consist with mRNA level (n = 5). **C** Rhythmic expression of core clock genes in control normal cells and klf7 knockdown cells (n = 4). Rhythmic expression of Clock was disrupted and the amplitude increased (n = 4). CT0 is the onset at hour 0 of subjective light period. The data are presented as the mean ± SEM. Statistical analysis (P^**^ < 0.01 and P^***^ < 0.001) between control group and knockdown group in (**A**, **B**) were performed by unpaired t test. Statistical analysis (P^*^ < 0.05, P^**^ < 0.01, P^***^ < 0.001 and P^****^ < 0.0001) between control group and klf7 knockdown group in (**C**) were performed by one-way ANOVA test

To obtain insight into the possible role of klf7 on Clock and other circadian genes of mammalian cells, we examined the expression changes of rhythm genes in klf7 knockdown cells. We knocked down klf7 with a lentiviral short hairpin RNA (shRNA) in N2A, and then found that Clock gene mRNA level and protein level were reduced (Fig. 3A, B). Due to the expression of Clock were decreased significantly in klf7 knockdown cells, we examined to see weather klf7 deficiency led to the abnormal circadian phenotypes. We synchronized these N2A cells and checked mRNA levels of circadian rhythm genes every four hours during the diurnal circadian cycle. We observed that Clock gene exhibited a rhythmic expression in normal cells and that this rhythmic expression was disrupted and even exhibited an inversion rhythm at some time points in klf7 knockdown cells. Moreover, the amplitude of Clock gene between the peak and nadir increased in a cycle (Fig. 3C), which is of an important factor in diurnal biology. Notably, abnormal rhythmic expression of Clock, as a core transcriptional regulatory protein in the circadian rhythm system, may lead to oscillations of downstream rhythm genes. To uncover this, we also assayed some other rhythm genes and found that the amplitudes of Nr1d1, Per2, Per3, Cry2 and Dbp gene were changed in a cycle and that the rhythm of Nr1d1 and Per2 were disturbed (Fig. 3C). These results suggested that klf7 deficiency made Clock exhibits an abnormal rhythmic expression and further triggered the oscillations of downstream rhythm genes. It has been shown that SNP in circadian rhythm genes is associated with ASD and attention deficit hyperactivity disorder [18, 30, 31]. The expression level of Clock gene was decreased in ASD individuals [32] and rs3762836 (p.H542R) located at Clock gene was found in Japanese ASD individuals [17]. In addition, 4q12 copy number variation including Clock gene have been associated with ASD [33, 34]. Interestingly, mutations in Clock direct transcription target Per1 and dimerization partner Npas2 are also associated with ASD [18, 35]. The preponderance of Clock involvement indicates that this transcription factor plays a variety of roles in regulating processes relevant to human health; some are linked to circadian rhythm and the others may be associated with ASD. This further supports our hypothesis that there is a link between circadian rhythm and klf7 and that klf7 can cause ASD by targeting Clock gene.

### klf7 deficiency mice show core autistic behavior

To examine if the circadian rhythm disruption can cause ASD in vivo, we constructed *Nestin-*Cre klf7 conditional knockout mice, in which exon 2 of klf7 is deleted under the control of loxp-Cre system. To examine whether klf7 deficient mice could reproduce autistic behavior as shown in klf7 mutations patients, we performed behavioral test on male WT mice and male klf7^−/−^ mice aged 2–3 months.

**Fig. 4:**
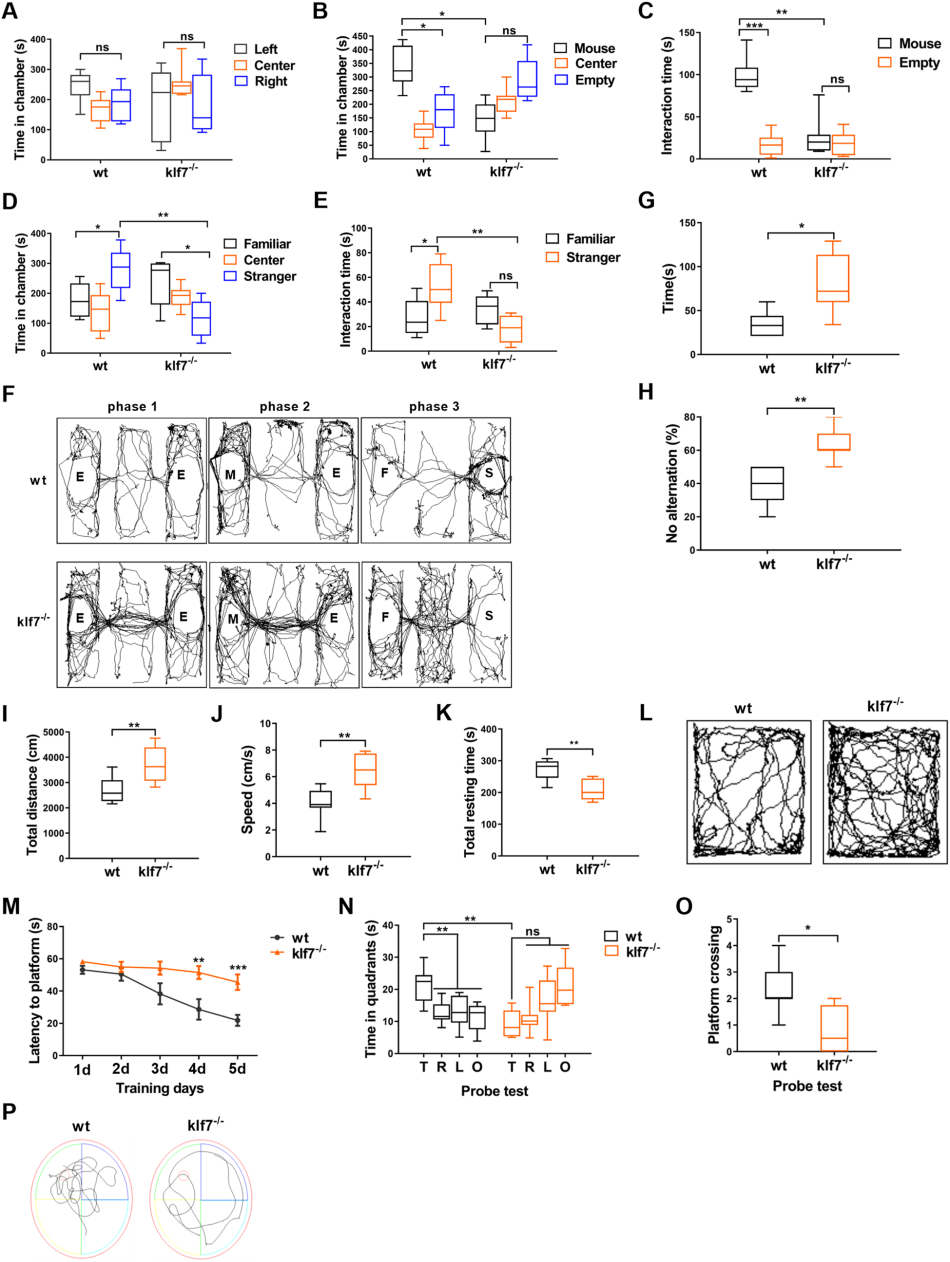
Klf7^−/−^ mice show autistic behavior. **A**–**F** Three chamber experiment. **A** WT mice (n = 10) and klf7^**−/−**^ mice (n = 8) showed no obvious preference for left and right chambers. **B** While WT mice (n = 10) spent more time in the chamber with a mouse, klf7^**−/−**^ mice (n = 8) didn’t show obvious preference for the chambers with and without a mouse. **C** Total interaction time spent on the chamber with a mouse and the empty chamber by klf7^**−/−**^ mice (n = 8) is equal, while the interaction time spent on the chamber with a mouse by klf7^**−/−**^ mice (n = 8) is significantly lower than that spent by WT mice (n = 10). **D** WT mice (n = 10) spent more time in the strange-mouse chamber, while klf7^**−/−**^ mice (n = 8) didn’t show preference for the strange-mouse chamber and familiar-mouse chamber. **E** Klf7^**−/−**^ mice (n = 8) spent significantly-less time interacting with a strange mouse as compared to WT mice (n = 10). **F** Representative movement tracks in three-chamber experiment. **G**, **H** Klf7^**−/−**^ mice (n = 8) showed obvious repetitive behaviors. **G** Klf7^−/−^ mice (n = 8) spent significantly-more time in self-grooming. **H** Klf7^**−/−**^ mice (n = 8) preferred the original target arm rather than exploring the novel target arm in the Y maze spontaneous selection experiment, as indicated by the ratio of entry into the original target arm in 10 trials. **I**–**L** Open field tests. **I** Klf7^−/−^ mice (n = 8) traveled significantly longer distances than WT mice (n = 10). **J** Klf7^−/−^ mice (n = 8) traveled significantly-faster than WT mice (n = 10). **K** Klf7^−/−^ mice (n = 8) spent significantly-shorter time on resting than WT mice (n = 10) did. **L** Representative tracks in the open field test. **M**–**P** Morris water maze test. **M** Spatial learning ability assessed by the latency to locate platform. Klf7^−/−^ mice (n = 8) spent more time on locating platform on the 5 training days, indicating impaired spatial learning ability. **N** In contrast to WT mice (n = 10) spending more time in the target region in the probe test, klf7^−/−^ mice (n = 8) didn’t explore the target region. T, target region; R, region to the right of the target right; L, region to the left of the target region; O, region opposite the target region. **O** WT mice (n = 10) crossed the platform more frequently than klf7^−/−^ mice (n = 8). **P** Representative tracks in the Morris water maze. The data are presented as the mean ± SEM. Statistical analysis (P^*^ < 0.05, P^**^ < 0.01, P^***^ < 0.001 and ns: no significance) in (**A**–**E**) and (**N**) were performed by two-way ANOVA test. Statistical analysis (P^*^ < 0.05 and P^**^ < 0.01) in (**G**–**H**, **I**–**K**) and (**O**) were performed by unpaired t test. Statistical analysis (P^**^ < 0.01 and P^***^ < 0.001) in (**M**) were performed by one-way ANOVA test

Three-chamber social interaction tests were performed to examine whether klf7 deficient mice had a core symptom of ASD—social deficit. WT mice and klf7^−/−^ mice didn’t show obvious preference for the left and right chambers during the adaptation phase (Fig. 4A). When the first strange mouse was introduced to a chamber, WT mice stayed in the mouse chamber significantly longer than that in the empty chamber. There was no significant difference in the time that klf7^−/−^ mice stayed in the mouse chamber and empty chamber, and klf7^−/−^ mice stayed in the mouse chamber significantly shorter than WT mice stayed in the mouse chamber (Fig. 4B, F). WT mice also spent significantly more time interacting with the mouse than that with the empty cage. However, klf7^−/−^ mice did not interact with the mouse compared with WT mice, the interaction time with mouse was significantly lower than that of WT mice (Fig. 4C). Upon introducing the second mouse to another chamber, WT mice preferred to stay in the second-mouse chamber rather than first-mouse chamber. Klf7^−/−^ mice did not show preference, and klf7^−/−^ mice stayed in the second mouse chamber or strange mouse chamber significantly shorter than WT mice (Fig. 4D, F). In terms of the interaction time, WT mice preferred to interact with the second or strange mouse rather than the first or familiar one. In contrast, the interaction time of klf7^−/−^ mice with the strange mouse and familiar mouse were equal, and the interaction time of klf7^−/−^ mice with the strange mouse was significantly lower than that of WT mice with the strange one (Fig. 4E). These results suggested that klf7 deficient mice had abnormal social behavior.

The self-grooming test and the Y-maze spontaneous selection test were performed to assess whether the klf7 deficient mice exhibit repetitive behaviors, which is also a core behavior of ASD. Our results showed that Klf7^−/−^ mice spent significantly more time on grooming than WT mice (Fig. 4G). In the Y maze spontaneous selection experiment, klf7^−/−^ mice preferred the first target arm and did not like to make a change, while WT mice preferred to explore the new arm (Fig. 4H). These results indicated that klf7 deletion mice had obvious repetitive behaviors.

In addition to the core behaviors of ASD, we also examined other symptoms associated with ASD. In the open field experiment, our results showed that the total distance that klf7^−/−^ mice had moved was significantly higher than that of the WT mice (Fig. 4I, L), and that their movement speeds were significantly increased (Fig. 4J) while their rest durations were also significantly reduced (Fig. 4K). In the Morris water maze test, klf7^−/−^ mice showed an obvious decreased learning ability during the five training days (Fig. 4M). During the testing period, WT mice spent more time in the target region while klf7^−/−^ mice didn’t explore the target region at all (Fig. 4N). Moreover, WT mice crossed the platform location more frequently while klf7^−/−^ mice swam freely (Fig. 4O, P). These data suggested that klf7^−/−^ mice had greater motor capacity, but were severely impaired spatial learning and memory function.

### Disrupting of Clock function in klf7^−/−^ mice

**Fig. 5:**
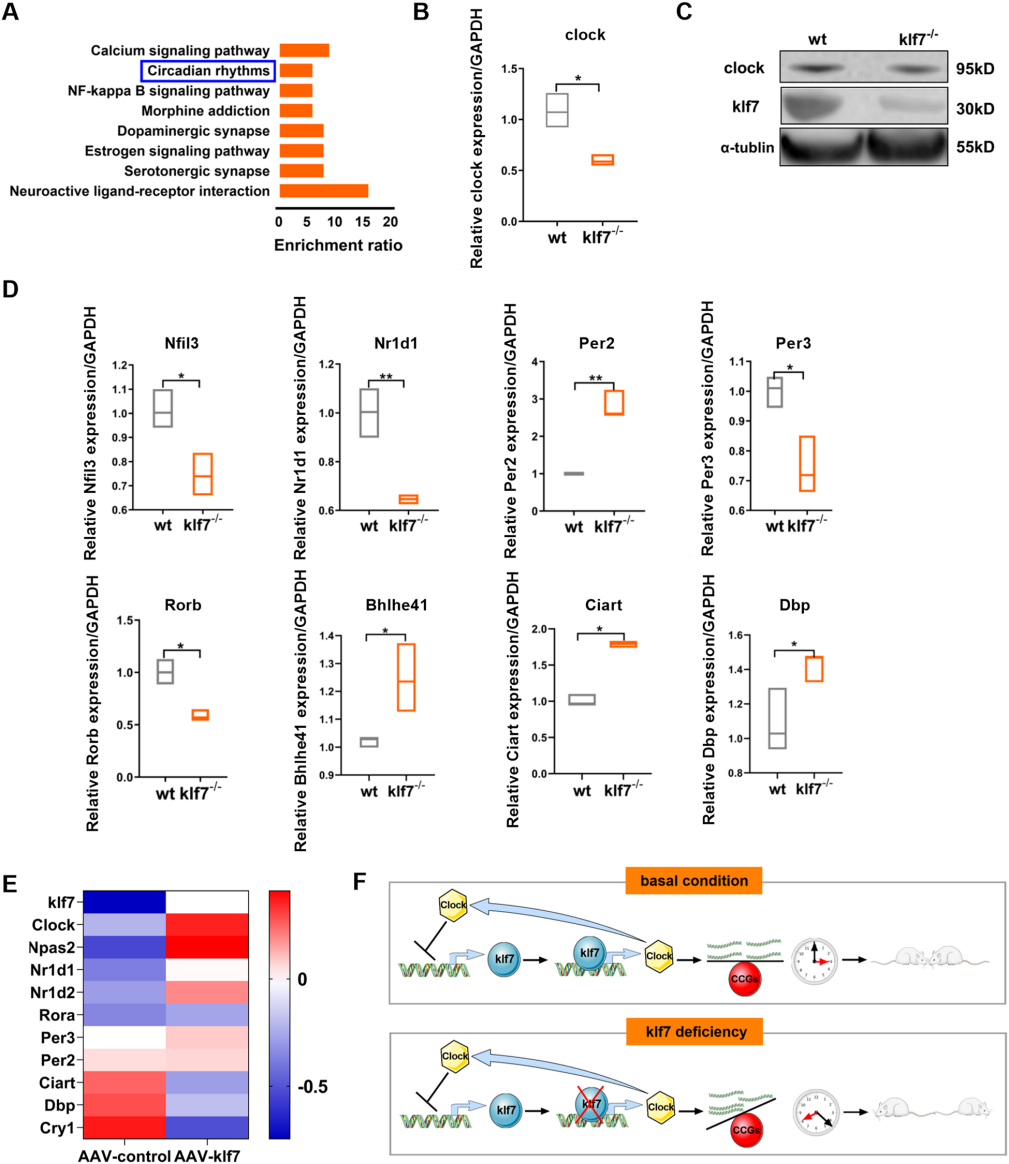
Disruption of clock function in klf7^−/−^ mice. **A** Functional enrichment analysis of differentially expressed genes (DEGs) in 7-day-old male klf7^**−/−**^ mice. These DEGs were mainly enriched in the processes related to circadian rhythms. **B** Clock mRNA levels measured by qPCR in klf7^**−/−**^ mice. **C** Clock protein levels measured by western blot in klf7^**−/−**^ mice. **D** Validation of the mRNA level of rhythm-related genes by qRT-PCR. **E** Graph showing the levels of rhythm-related genes, which were restored by administering adeno-associated virus (AAV) mediated overexpression of klf7 in klf7^±^ mice. **F** Proposed model of klf7’s activity on regulating circadian rhythm genes in ASD development. Under the basal condition, klf7 can target Clock gene and indirectly regulate downstream rhythmic genes, thus forming a feedback loop with Clock gene to maintain the stability of circadian rhythm system. When klf7 is deficient, dysregulation of circadian rhythm gene disrupts the circadian system and leads to ASD. The data are presented as the mean ± SEM. Statistical analysis (P^*^ < 0.05 and P^**^ < 0.01) in (**B**) and (**D**) were performed by unpaired t test

To determine whether klf7 deficiency can affect circadian rhythms in vivo, we performed the RNA-seq on 7-day-old male WT mice and klf7^**−/−**^ mice. KEGG enrichment analysis was performed on the differentially expressed genes (DEGs), and the results showed that DEGs were significantly enriched in circadian rhythms (Fig. 5A). Regarding the effect of klf7 on Clock gene, our quantitative PCR results showed the reduced Clock level (Fig. 5B). Clock protein level was also reduced, being consistent with the RNA level (Fig. 5C). To examine whether the decrease of Clock protein could cause the downstream rhythm genes abnormal, we detected the expression levels of other important rhythm genes in the brain of klf7^**−/−**^ mice, and found that the expression levels of other rhythm genes were also disturbed (Fig. 5D). In our previous study [10], klf7^**±**^ mice showed the mild autistic behavior, which could be improved if klf7 is expressed in the brain by the adeno-associated virus. So we also performed the RNA-seq on klf7^**±**^ mice treated with adeno-associated virus, and analyzed the expression level of rhythm genes in the brain of klf7^**±**^ mice before and after treatment. Our results showed that increased klf7 level could reverse the expression level of rhythm genes (Fig. 5E) and rescue the autistic behavior [10]. These results suggest that klf7 is an important member involved in the regulation of rhythmic circadian genes in vivo and links circadian rhythms to ASD (Fig. 5F).

### Melatonin regulates the expression of rhythm genes and restores the core autistic behavior

**Fig. 6:**
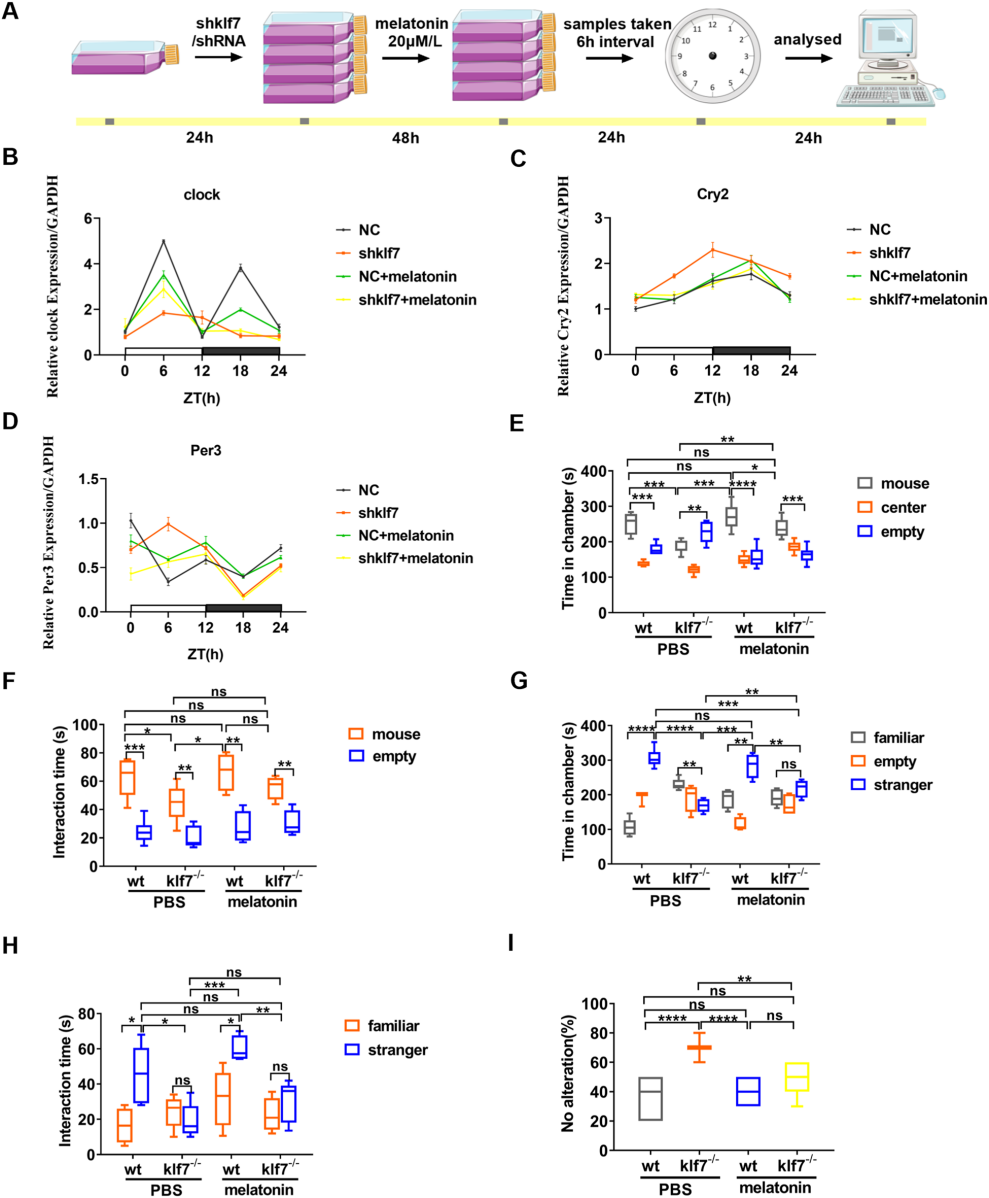
Melatonin rescues social deficits and repetitive behavior in klf7^±^ mice. **A** Schematic diagram of detection of melatonin regulation effect on circadian rhythm in N2A cells. **B** qPCR analysis of Clock gene expression in N2A cells before and after melatonin incubation (n = 4). **C** qPCR analysis of Cry2 gene expression in N2A cells before and after melatonin incubation (n = 4). **D** qPCR analysis of Per3 gene expression in N2A cells before and after melatonin incubation (n = 4). **E** Histogram showing the amount of time spent in both the chamber with an interactive mouse and the empty cage by PBS-treated WT mice (n = 10), PBS-treated klf7^−/−^ mice (n = 10), melatonin-treated WT mice (n = 10) and melatonin-treated klf7^−/−^ mice (n = 10) in the three-chambers-social-preference experiment. Whereas PBS-treated klf7^−/−^ mice did not show an obvious social preference, melatonin-treated klf7^−/−^ mice showed improved social ability. **F** Histogram showing the amount of time spent with the interactive mouse or in the empty cage by PBS-treated WT mice (n = 10), PBS-treated klf7^−/−^ mice (n = 10), melatonin-treated WT mice (n = 10) and melatonin-treated klf7^−/−^ mice (n = 10) in the three-chambers-social-preference experiment. **G** Histogram showing the amount of time spent in the chambers with a strange mouse or a familiar mouse by PBS-treated WT mice (n = 10), PBS-treated klf7^−/−^ mice (n = 10), melatonin-treated WT mice (n = 10) and melatonin-treated klf7^−/−^ mice (n = 10) in the three-chamber-social-preference experiment. **H** Histogram showing the amount of time spent with a strange mouse or a familiar mouse by PBS-treated WT mice (n = 10), PBS-treated klf7^−/−^ mice (n = 10), melatonin-treated WT mice (n = 10) and melatonin-treated klf7^−/−^ mice (n = 10) in the three-chamber-social-preference experiment. **I** Histogram showing the proportion of PBS-treated WT mice (n = 10), PBS-treated klf7^−/−^ mice (n = 10), melatonin-treated WT mice (n = 10) and melatonin-treated klf7^−/−^ mice (n = 10) sticking to the original choice in 10 times Y maze spontaneous selection experiments. The data are presented as the mean ± SEM. Statistical analysis (P^*^ < 0.05, P^**^ < 0.01, P^***^ < 0.001, P^****^ < 0.0001 and ns: no significance) in (**E**–**H**) were performed by two-way ANOVA test. Statistical analysis (P^**^ < 0.01, P^****^ < 0.0001 and ns: no significance) in (**I**) were performed by one-way ANOVA test

Based on the above-discovered relationship among the ASD, circadian rhythms and klf7, we further examined whether restoring circadian rhythms could be used to treat ASD. Melatonin is an endogenous neurohormone synthesized by the pineal gland, and its main role includes regulating circadian rhythm [36, 37]. Previous in vivo studies have shown that melatonin is involved in regulating the expression of several clock genes in central and peripheral system, such as Clock, Per1, Per2, Bmal1, Rev-ERba and Cry1 [38, 39]. As discussed above, klf7 deficiency disrupted the rhythmic expression of Clock and other rhythmic genes. In this study we subsequently examined whether melatonin could restore the circadian rhythm disorder induced by klf7 deletion and rescue the autistic behavior of klf7^−/−^ mice. For this, we first examined its regulatory effect on rhythm disorders in N2A cells. Upon 24 h of subculture, N2A cells were added with shklf7 or control shRNA lentivirus infection for 48 h, then 20 μm/L melatonin were added into medium and incubated for 24 h. Samples were taken every 6 h, and then the effect of melatonin on the expression level of rhythm genes was analyzed (Fig. 6A). Clock gene showed obvious rhythmic expression in the normal cells and this rhythm was disturbed in the shklf7 group. Once melatonin was introduced and incubated, Clock gene was still rhythmically expressed in the normal cells, but with the reduced amplitude. Also, Clock gene was rhythmically expressed in shklf7 group with melatonin but the amplitude was lower than that of the normal cells (Fig. 6B). The regulation of Clock gene expression level and rhythmic oscillation caused by melatonin brought our attention to Cry and Per, both of which are downstream target genes of Clock. Our results in this regard showed that melatonin could restore the rhythmic expression of Cry2 gene (Fig. 6C) and regulate the oscillation amplitude of Per3 (Fig. 6D). Our data demonstrate that melatonin can regulate the expression and oscillation of rhythm genes and maintain the normal rhythm system.

Also, we were interested in whether it could restore abnormal behavior in mice. Mice were intraperitoneally injected with melatonin at 10 mg/kg for 15 days, followed by behavioral analysis. The tested mice were acclimated in the equipment for 10 min, and then a male mouse of the same age, who had not been reared with tested mice in the same cage, was randomly introduced into the left and right chambers. In the PBS injection group, the time of WT mice staying in the interactive mouse chamber was significantly higher than that in the empty cage chamber, while the time of klf7^−/−^ mice staying in the interactive mouse chamber was significantly lower than that in the empty cage chamber, which is also significantly lower than that of WT mice staying in the interactive mouse chamber. In the melatonin injection group, the time of WT mice staying in the interactive mouse chamber was significantly higher than that in the empty cage chamber. The time of melatonin treated klf7^−/−^ mice staying in the interactive mouse chamber was significantly higher than that in empty cage chamber, although it was significantly lower than that of melatonin treated WT mice staying in the interactive mouse chamber, but significantly higher than PBS treated klf7^−/−^ mice staying in the interactive mouse chamber (Fig. 6E). The interaction time of PBS-treated WT mice with interactive mice was significantly higher than that in the empty cage, while the interaction time of PBS-treated klf7^−/−^ mice with interactive mice was significantly higher than that in empty cage, but significantly lower than that of WT mice with the interactive mice. After melatonin treatment, the interaction time between WT mice and the interactive mice was significantly higher than that in the empty cage. Melatonin-treated klf7^−/−^ mice had significantly longer interaction time with the interactive mice than in the empty cage, which wasn’t significantly different from the wild-type mice (Fig. 6F).

When the second strange mouse was introduced, PBS-treated WT mice preferred to stay in this strange mouse chamber rather than familiar mouse chamber, while PBS-treated klf7^−/−^ mice spent significantly less time in the strange mouse chamber than that in the familiar mouse chamber. The time spent in the strange mouse chamber by PBS-treated klf7^−/−^ mice was also significantly lower than the time that PBS-treated WT mice stayed in the strange mouse chamber. Melatonin-treated WT mice spent significantly more time in the strange mouse chamber than in the familiar mouse chamber. The time of melatonin-treated klf7^−/−^ mice in the strange mouse chamber was not different from that in the familiar mouse chamber, which was significantly lower than that melatonin-treated WT mice spent in the strange mouse chamber, but significantly higher than that PBS-treated klf7^−/−^ mice spent in the strange mouse chamber (Fig. 6G). The interaction time spent by PBS-treated WT mice with the strange mouse was significantly higher than that spent with the familiar mouse, while the interaction time spent by PBS-treated klf7^−/−^ mice with the strange mouse and familiar mouse was equal. The interaction time between PBS-treated klf7^−/−^ mice and the strange mouse was significantly lower than that between PBS-treated WT mice and the strange mouse. The melatonin-treated WT mice still preferred to interact with the strange mouse, but melatonin-treated klf7^−/−^ mice still spent equal interaction time between the strange mouse and familiar mouse, and there is no significant difference between interaction time spent by melatonin-treated klf7^−/−^ mice and PBS-treated klf7^−/−^ mice (Fig. 6H). These data suggest that melatonin can provide some degree of improvement from social deficits.

In the Y maze spontaneous selection experiment, the proportion of PBS-treated klf7^−/−^ mice sticking to the original choice was significantly higher than that of WT mice, and the probability of melatonin-treated klf7^−/−^ mice was significantly lower than that after treatment, suggesting that melatonin can help to reduce the repetitive stereotypical behavior of mice (Fig. 6I). These results suggest that circadian rhythm play a significant role in the development of ASD and likely, is a relevant target for the development of therapeutic strategy in ASD.

## Discussion

In our study, we found that klf7 can target rhythmic gene and form a feedback loop with Clock gene. Klf7 deficiency not only caused N2A cells to produce dysregulated circadian phenotypes, but also led to the autistic behavior in mice. Klf7 deficiency leads to the development of ASD and restore circadian rhythms can rescue the autistic behavior, indicating that circadian rhythm is an important cause for ASD. Our study tested and supported the hypotheses that there was a link among klf7, circadian rhythms, and ASD.

Klf7 is a rhythm transcription factor that can enrich rhythm genes in the suprachiasmatic nucleus (SCNs) and regulate the expression of rhythm genes with clock transcription factors [11, 12]. Our previous study also found that klf7 target genes were also significantly enriched in the circadian rhythm pathway [10]. These evidences indicate that klf7 is involved in the regulation of circadian rhythm and may play an important role in circadian activities. Circadian rhythm controls various biological processes in living organisms; impaired circadian clock networks or rhythm function can increase susceptibility to psychiatric disorders, such as ASD [15]. Recent studies on the cognitive and developmental psychology have shown the important role of rhythms in the development of early social interaction [40]. Meanwhile, other studies have also linked ASD to circadian disturbances [41, 42]. This suggests that there is an implication between circadian rhythms and ASD etiology. Klf7 deficiency leads to the circadian dysregulation, implicating that klf7 may be one link between circadian rhythm and ASD.

Clock gene is a core clock transcription factor to drive the daily transcription patterns of some clock-controlled genes (CCGs) in some tissues. Studies have shown that Clock dysregulation can disrupt the gene co-expression network related to neurological diseases and play a role in controlling the transcriptional cascade in human brain evolution [43]. Clock gene mutation can cause a lengthened rest-activity cycle [44]. Research has shown that polymorphism (rs1801260/rs3762836) of Clock gene is associated with attention deficit hyperactivity disorder (ADHA) and ASD [17, 30, 31, 45–47], being consistent with the performance of klf7^−/−^ mice in open field experiment as observed in the present study. Moreover, decreased Clock level was coincided with the results reported from ASD individuals [48]. A more recent study has been in support of the proposed association between Clock gene and ASD [17]. In the present study, we tested and proved the relationship between Clock gene and ASD in vivo for the first time and demonstrated that the decreased Clock level also led to abnormalities of its downstream rhythm genes. Abnormalities in rhythm genes have been associated with memory in mice and communication timing in fruit flies [20, 49]. All of evidences further support that circadian rhythm abnormality is a cause of ASD. Clock gene, as a core transcriptional regulator of rhythm genes, is a major factor in ASD development.

Circadian rhythm dysregulation is associated with many diseases, adjusting circadian rhythm is a promising method in the treatment of diseases [50, 51]. In the light of the demonstrated relationship between ASD and circadian rhythm, we also examined the treatment of ASD by adjusting circadian rhythms. Melatonin is a circadian synchronizer [52] and is involved in regulating circadian expression of Per1, Per2, Bmal1, Clock and Cry1 in central and peripheral tissues [38, 39]. Melatonin can synchronizes circadian oscillations by affecting circadian expression of Per1 and Bmal1 in the cardiovascular system of rat heart [53], as well as the metabolism and hormonal function by regulating Per2, Clock and Rev-ERba in adipose tissue [54, 55]. Taken together, these studies demonstrate the important role of melatonin in regulating the expression of rhythmic genes and synchronization of central oscillators. In the present study, we illustrate that melatonin can regulate the expression level and amplitude of Clock gene and its downstream target rhythm genes induced by klf7 deficiency. Meanwhile, melatonin can be used to alleviate the core symptoms of ASD in klf7^−/−^ mice, further supporting the link of klf7 to ASD through the regulation of the circadian genes. The role of melatonin in regulating circadian rhythms makes it a good candidate of drugs for ASD treatment.

The limitation of this study is that there are no detailed evidences to explain how the feedback loop between klf7 and Clock is generated, which would be interesting for future research.

## Conclusions

Klf7 has been recently identified as a causal gene for autism spectrum disorder, but leaving a lot to be discovered. Our findings in the present study demonstrate that klf7 causes ASD by targeting Clock genes that trigger circadian genes dysregulated and circadian oscillation. In the light of a strong link between dysregulated circadian rhythm and ASD, our study also supports that circadian rhythm may be a relevant target for the development of therapeutic strategy in ASD.

## Methods

### Mice and drug treatment

Homozygous klf7^loxp/loxp^ mice were purchased from Cyagen Biosciences Inc. (Suzhou, China), with loxp sites inserted into exon 2. Klf7^−/−^ mice were constructed by crossing homozygous klf7^loxp/loxp^ mice with mice expressing *Cre.* Littermates WT mice were used as control for klf7^−/−^ mice. All mice were placed in a temperature-controlled environment under the cycle of 12 h light and 12 h dark. All animal husbandry and laboratory procedures were approved by Harbin Institute of Technology Laboratory Animal Welfare Ethics Committee (IACUC-2020036). In our study, male mice of 2–3 months old were used for behavioral experiments (either with or without drug treatment). Melatonin treatment (10 mg/kg body weight) [56, 57] or PBS was injected via intraperitoneal injection, once a day over 15 days. Mice were randomly grouped into the melatonin group or PBS group and the examination were blinded to genotypes. Once behavioral tests were completed, the mice were used in the subsequent experiments as described below.

### ChIP-seq analysis

ChIP-seq was performed as described previously [58]. Briefly, the cells were crosslinked with 1% formaldehyde for 10 min and neutralized with glycine (150 mM final solution). Chromatin was cleaved into about 300 bp fragments by ultrasonic treatment after cells were lysed. Chromatin fragments were incubated with antibody (Abcam, ab9110; Abcam, ab172730) overnight at 4 °C, followed by incubating with magnetic beads for 2 h at 4 °C. The proteins were eluted and reversed crosslinking overnight at 65 °C. DNA was extracted with Phenol–chloroform for sequencing. The purified DNA were pair-end sequenced on an illumina platform (Illumina, CA, USA).

Clean reads were aligned to the mouse reference genome (Ensemble_GRCm38.94) using BWA mem (v 0.7.12). IP enrichment regions were identified using MACS2 (version 2.1.0) peak calling software. A q-value threshold of 0.05 was used to identify significant peaks of klf7, which was defined as the overlapping peaks between two biological ChIP-seq replications.

### Luciferase reporter assay

293 T cells were transfected with PIRM-control or PIRM-klf7 vector and PGL4-Clock vector at a concentration of 1000 ng/per well. Cells were lysed with 100 μL cold 1 × PLB lysis buffer (Promega), which were then shaken for 15 min at room temperature. The lysis was centrifuged at 12,000 rpm for 2 min and 20 μL supernatant was used for luciferase activity assay (Promega).

### RNA-seq analysis

RNA-seq was performed as described previously [58]. Total RNA was extracted from klf7 knockdown organoids and control organoids, whole brains of 7-day-old male WT mice and 7-day-old male klf7^−/−^ mice, whole brains of klf7^±^ mice injected with AAV-NC virus and AAV-klf7 virus. The whole brains of 4 mice per genotype and 20 organoids per group were considered as one biological replication.

Hisat2 (v2.0.5) was used to align reads to the human genome (homo_sapiens_grch38_p12) and the mouse genome (mus_musculus_grcm38_p6). The featureCounts v1.5.0-p3 was used to count the reading numbers mapped to each gene; and FPKM of each gene was calculated based on the length of the gene and reading number mapped to this gene. The DESeq2 R package (1.20.0) was used to analyze differential expression gene, where padj < 0.01 and |log2(foldchange)|> 1 were used to detect significantly differential expression genes. ClusterProfiler R package (3.8.1) was used to test the statistical enrichment of differential expression genes in KEGG pathways.

### Klf7 knockdown

N2A cells were used to study klf7 knockdown, 20 μL (1.0 × 10^5^ infectious units of virus) of either validated shklf7 or negative control shRNA lentivirus were added to the cells. After 48 h, qRT-PCR and western blot were performed to examine the knockout efficiency.

### Three-chamber social experiment

The social experiment was performed as previously described [59] with minor modifications. Male mice of 2–3 months old were used and placed in a testing room for 2 h prior to the behavioral test. After that, the mice to be tested were placed in an apparatus for 10 min for habituation. When a strange mouse was introduced to a wire cage in one side of chambers, the tested mice were allowed to explore all three-chambers for 10 min. Then another strange mouse was placed in the empty cage and the tested mice were allowed to explore all three-chambers for 10 min again. The time duration that the tested mice spent in each chamber and the time duration spent on interacting with the first- and second-introduced mice were recorded for examination.

### Self-grooming

Self-grooming behaviors were tested as previous study [59] with minor modifications. The mice were first conditioned in the testing device for 10 min and then examined in terms of the total self-grooming time over the next 10 min.

### Y maze spontaneous selection experiment

The selection experiment was performed as previous study [60] with minor modification. The tested mice were placed in the middle arm, allowing to select between the left and right arms to enter. The tested mice were left in the arms selected by themselves for 10 min. Next, the number of mice entered in the originally-selected arm during 10 trials was recorded for examination.

### Open field test

The open field test was performed as previous study [61]. The tested mice were allowed to explore apparatus for 10 min and their activities were recorded by a camera for another period of 10 min.

### Morris water maze test

Morris water maze test was performed as previously described in the study [59] with few modifications. Each mouse was allowed to perform 4 trials a day over 5 days. The time to locate the platform was recorded during the training period. In the testing trial, after removing the platform each tested mouse was allow to search for the platform in 60 s. The time spent in each quadrant and the number of crossing the platform location were recorded.

### Statistical analysis

All data were presented as the mean ± SEM and analyzed by unpaired t test, one-way ANOVA test and two-way ANOVA test.

## Data Availability

RNA-seq data and ChIP-seq data that supporting the conclusions of this study are available on zenodo web site (https://zenodo.org/). DOI number for RNA-seq data of 1 month old wild type mice and 1 month old klf7^±^ mice is 5236120 (https://zenodo.org/search?page=1&size=20&q=5236120). DOI number for RNA-seq data of klf7^±^/AAV-control mice and klf7^±^/AAV-klf7 mice is 5242635 (https://zenodo.org/search?page=1&size=20&q=5242635). DOI number for RNA-seq data of klf7 knockdown human brain organoid model is 5242821 (https://zenodo.org/search?page=1&size=20&q=5242821). DOI number for ChIP-seq data of klf7 in N2A cells is 5243430 (https://zenodo.org/search?page=1&size=20&q=5243430).

## Abbreviations

Klf7: Krüppel-like factor 7
ASD: Autism spectrum disorder
CCGs: Clock-controlled genes
ShRNA: Short hairpin RNA
DEGs: Differentially expressed genes
SCNs: Suprachiasmatic nucleus
ADHA: Attention deficit hyperactivity disorder
WT: Wild type

## References

[1] Laub F, Lei L, Sumiyoshi H, Kajimura D, Dragomir C, Smaldone S (2005). Transcription factor KLF7 is important for neuronal morphogenesis in selected regions of the nervous system. Mol Cell Biol.

[2] Courtens W, Speleman F, Messiaen L, Bormans J, Van Roy N, Vamos E (1997). Interstitial deletion 2q33.3-q34 in a boy with a phenotype resembling the Seckel syndrome. Am J Med Genet.

[3] Pescucci C, Meloni I, Bruttini M, Ariani F, Longo I, Mari F (2003). Chromosome 2 deletion encompassing the MAP2 gene in a patient with autism and Rett-like features. Clin Genet.

[4] Bisgaard AM, Kirchhoff M, Tumer Z, Jepsen B, Brondum-Nielsen K, Cohen M (2006). Additional chromosomal abnormalities in patients with a previously detected abnormal karyotype, mental retardation, and dysmorphic features. Am J Med Genet A.

[5] Brandau DT, Lund M, Cooley LD, Sanger WG, Butler MG (2008). Autistic and dysmorphic features associated with a submicroscopic 2q33.3-q34 interstitial deletion detected by array comparative genomic hybridization. Am J Med Genet A.

[6] Jang DH, Chae H, Kim M (2015). Autistic and Rett-like features associated with 2q33.3-q34 interstitial deletion. Am J Med Genet A.

[7] Powis Z, Hart A, Cherny S, Petrik I, Palmaer E, Tang S (2017). Clinical diagnostic exome evaluation for an infant with a lethal disorder: genetic diagnosis of TARP syndrome and expansion of the phenotype in a patient with a newly reported RBM10 alteration. BMC Med Genet.

[8] Yuen RK, Merico D, Cao H, Pellecchia G, Alipanahi B, Thiruvahindrapuram B (2016). Genome-wide characteristics of de novo mutations in autism. NPJ Genom Med.

[9] Turner TN, Coe BP, Dickel DE, Hoekzema K, Nelson BJ, Zody MC (2017). Genomic patterns of de novo mutation in simplex autism. Cell.

[10] Tian H, Qiao S, Zhao Y, Jin X, Wang C, Wang R (2022). Kruppel-like transcription factor 7 is a causal gene in autism development. Int J Mol Sci.

[11] Wen S, Ma D, Zhao M, Xie L, Wu Q, Gou L (2020). Spatiotemporal single-cell analysis of gene expression in the mouse suprachiasmatic nucleus. Nat Neurosci.

[12] Cheng AH, Bouchard-Cannon P, Hegazi S, Lowden C, Fung SW, Chiang CK (2019). SOX2-dependent transcription in Clock neurons promotes the robustness of the central circadian pacemaker. Cell Rep.

[13] Tordjman S, Anderson GM, Bellissant E, Botbol M, Charbuy H, Camus F (2012). Day and nighttime excretion of 6-sulphatoxymelatonin in adolescents and young adults with autistic disorder. Psychoneuroendocrinology.

[14] Yenen AS, Cak HT (2020). Melatonin and circadian rhythm in autism spectrum disorders. Turk Psikiyatri Derg.

[15] Charrier A, Olliac B, Roubertoux P, Tordjman S (2017). Clock genes and altered sleep-wake rhythms: their role in the development of psychiatric disorders. Int J Mol Sci.

[16] Takahashi JS, Hong HK, Ko CH, McDearmon EL (2008). The genetics of mammalian circadian order and disorder: implications for physiology and disease. Nat Rev Genet.

[17] Yang Z, Matsumoto A, Nakayama K, Jimbo EF, Kojima K, Nagata K (2016). Circadian-relevant genes are highly polymorphic in autism spectrum disorder patients. Brain Dev.

[18] Nicholas B, Rudrasingham V, Nash S, Kirov G, Owen MJ, Wimpory DC (2007). Association of Per1 and Npas2 with autistic disorder: support for the clock genes/social timing hypothesis. Mol Psychiatry.

[19] Toh KL, Jones CR, He Y, Eide EJ, Hinz WA, Virshup DM (2001). An hPer2 phosphorylation site mutation in familial advanced sleep phase syndrome. Science.

[20] Sakai T, Tamura T, Kitamoto T, Kidokoro Y (2004). A clock gene, period, plays a key role in long-term memory formation in Drosophila. Proc Natl Acad Sci U S A.

[21] Janich P, Pascual G, Merlos-Suarez A, Batlle E, Ripperger J, Albrecht U (2011). The circadian molecular clock creates epidermal stem cell heterogeneity. Nature.

[22] Marcheva B, Ramsey KM, Buhr ED, Kobayashi Y, Su H, Ko CH (2010). Disruption of the clock components CLOCK and BMAL1 leads to hypoinsulinaemia and diabetes. Nature.

[23] Wimpory D, Nicholas B, Nash S (2002). Social timing, clock genes and autism: a new hypothesis. J Intellect Disabil Res.

[24] Liu D, Nanclares C, Simbriger K, Fang K, Lorsung E, Le N (2022). Autistic-like behavior and cerebellar dysfunction in Bmal1 mutant mice ameliorated by mTORC1 inhibition. Mol Psychiatry.

[25] Mertens J, Marchetto MC, Bardy C, Gage FH (2016). Evaluating cell reprogramming, differentiation and conversion technologies in neuroscience. Nat Rev Neurosci.

[26] Pasca SP (2018). The rise of three-dimensional human brain cultures. Nature.

[27] Gordon A, Yoon SJ, Tran SS, Makinson CD, Park JY, Andersen J (2021). Long-term maturation of human cortical organoids matches key early postnatal transitions. Nat Neurosci.

[28] Takahashi JS (2015). Molecular components of the circadian clock in mammals. Diabetes Obes Metab.

[29] Reppert SM, Weaver DR (2002). Coordination of circadian timing in mammals. Nature.

[30] Kissling C, Retz W, Wiemann S, Coogan AN, Clement RM, Hunnerkopf R (2008). A polymorphism at the 3'-untranslated region of the CLOCK gene is associated with adult attention-deficit hyperactivity disorder. Am J Med Genet B Neuropsychiatr Genet.

[31] Xu X, Breen G, Chen CK, Huang YS, Wu YY, Asherson P (2010). Association study between a polymorphism at the 3'-untranslated region of CLOCK gene and attention deficit hyperactivity disorder. Behav Brain Funct.

[32] Hu VW, Sarachana T, Kim KS, Nguyen A, Kulkarni S, Steinberg ME (2009). Gene expression profiling differentiates autism case-controls and phenotypic variants of autism spectrum disorders: evidence for circadian rhythm dysfunction in severe autism. Autism Res.

[33] Sarachana T, Zhou R, Chen G, Manji HK, Hu VW (2010). Investigation of post-transcriptional gene regulatory networks associated with autism spectrum disorders by microRNA expression profiling of lymphoblastoid cell lines. Genome Med.

[34] Griswold AJ, Ma D, Cukier HN, Nations LD, Schmidt MA, Chung RH (2012). Evaluation of copy number variations reveals novel candidate genes in autism spectrum disorder-associated pathways. Hum Mol Genet.

[35] Neale BM, Kou Y, Liu L, Ma'ayan A, Samocha KE, Sabo A (2012). Patterns and rates of exonic de novo mutations in autism spectrum disorders. Nature.

[36] Reiter RJ, Calvo JR, Karbownik M, Qi W, Tan DX (2000). Melatonin and its relation to the immune system and inflammation. Ann N Y Acad Sci.

[37] Pundir M, Papagerakis S, De Rosa MC, Chronis N, Kurabayashi K, Abdulmawjood S (2022). Emerging biotechnologies for evaluating disruption of stress, sleep, and circadian rhythm mechanism using aptamer-based detection of salivary biomarkers. Biotechnol Adv.

[38] Dardente H, Menet JS, Poirel VJ, Streicher D, Gauer F, Vivien-Roels B (2003). Melatonin induces Cry1 expression in the pars tuberalis of the rat. Brain Res Mol Brain Res.

[39] von Gall C, Weaver DR, Moek J, Jilg A, Stehle JH, Korf HW (2005). Melatonin plays a crucial role in the regulation of rhythmic clock gene expression in the mouse pars tuberalis. Ann N Y Acad Sci.

[40] Tordjman S, Najjar I, Bellissant E, Anderson GM, Barburoth M, Cohen D (2013). Advances in the research of melatonin in autism spectrum disorders: literature review and new perspectives. Int J Mol Sci.

[41] Cortesi F, Giannotti F, Ivanenko A, Johnson K (2010). Sleep in children with autistic spectrum disorder. Sleep Med.

[42] Richdale AL, Schreck KA (2009). Sleep problems in autism spectrum disorders: prevalence, nature, and possible biopsychosocial aetiologies. Sleep Med Rev.

[43] Fontenot MR, Berto S, Liu Y, Werthmann G, Douglas C, Usui N (2017). Novel transcriptional networks regulated by CLOCK in human neurons. Genes Dev.

[44] Vitaterna MH, King DP, Chang AM, Kornhauser JM, Lowrey PL, McDonald JD (1994). Mutagenesis and mapping of a mouse gene, Clock, essential for circadian behavior. Science.

[45] Serretti A, Benedetti F, Mandelli L, Lorenzi C, Pirovano A, Colombo C (2003). Genetic dissection of psychopathological symptoms: insomnia in mood disorders and CLOCK gene polymorphism. Am J Med Genet B Neuropsychiatr Genet.

[46] Ozburn AR, Purohit K, Parekh PK, Kaplan GN, Falcon E, Mukherjee S (2016). Functional implications of the CLOCK 3111T/C single-nucleotide polymorphism. Front Psychiatry.

[47] Shi SQ, White MJ, Borsetti HM, Pendergast JS, Hida A, Ciarleglio CM (2016). Molecular analyses of circadian gene variants reveal sex-dependent links between depression and clocks. Transl Psychiatry.

[48] Huang MC, Ho CW, Chen CH, Liu SC, Chen CC, Leu SJ (2010). Reduced expression of circadian clock genes in male alcoholic patients. Alcohol Clin Exp Res.

[49] Garcia JA, Zhang D, Estill SJ, Michnoff C, Rutter J, Reick M (2000). Impaired cued and contextual memory in NPAS2-deficient mice. Science.

[50] Ruan W, Yuan X, Eltzschig HK (2021). Circadian rhythm as a therapeutic target. Nat Rev Drug Discov.

[51] Lee Y (2021). Roles of circadian clocks in cancer pathogenesis and treatment. Exp Mol Med.

[52] Pevet P, Challet E (2011). Melatonin: both master clock output and internal time-giver in the circadian clocks network. J Physiol Paris.

[53] Zeman M, Herichova I (2013). Melatonin and clock genes expression in the cardiovascular system. Front Biosci (Schol Ed).

[54] Kennaway DJ, Owens JA, Voultsios A, Wight N (2012). Adipokines and adipocyte function in Clock mutant mice that retain melatonin rhythmicity. Obesity (Silver Spring).

[55] Delezie J, Dumont S, Dardente H, Oudart H, Grechez-Cassiau A, Klosen P (2012). The nuclear receptor REV-ERBalpha is required for the daily balance of carbohydrate and lipid metabolism. FASEB J.

[56] Wang HB, Tahara Y, Luk SHC, Kim YS, Hitchcock ON, MacDowell Kaswan ZA (2020). Melatonin treatment of repetitive behavioral deficits in the Cntnap2 mouse model of autism spectrum disorder. Neurobiol Dis.

[57] Wang Q, Zhu D, Ping S, Li C, Pang K, Zhu S (2020). Melatonin recovers sleep phase delayed by MK-801 through the melatonin MT2 receptor- Ca(2+) -CaMKII-CREB pathway in the ventrolateral preoptic nucleus. J Pineal Res.

[58] Odawara J, Harada A, Yoshimi T, Maehara K, Tachibana T, Okada S (2011). The classification of mRNA expression levels by the phosphorylation state of RNAPII CTD based on a combined genome-wide approach. BMC Genomics.

[59] Peca J, Feliciano C, Ting JT, Wang W, Wells MF, Venkatraman TN (2011). Shank3 mutant mice display autistic-like behaviours and striatal dysfunction. Nature.

[60] Shoji H, Hagihara H, Takao K, Hattori S, Miyakawa T (2012). T-maze forced alternation and left-right discrimination tasks for assessing working and reference memory in mice. J Vis Exp.

[61] Katayama Y, Nishiyama M, Shoji H, Ohkawa Y, Kawamura A, Sato T (2016). CHD8 haploinsufficiency results in autistic-like phenotypes in mice. Nature.

